# Proton beam therapy for muscle-invasive bladder cancer: A systematic review and analysis with Proton-Net, a multicenter prospective patient registry database

**DOI:** 10.1093/jrr/rrad027

**Published:** 2023-04-25

**Authors:** Masayuki Araya, Hitoshi Ishikawa, Kentaro Nishioka, Kazushi Maruo, Hirofumi Asakura, Takashi Iizumi, Masaru Takagi, Masao Murakami, Haruhito Azuma, Wataru Obara, Hidefumi Aoyama, Hideyuki Sakurai

**Affiliations:** Proton Therapy Centre, Aizawa Hospital, 2-5-1, Honjo, Matsumoto, Nagano 390-8510, Japan; National Institutes for Quantum Science and Technology, QST Hospital, 4-9-1, Anagawa, Inage, Chiba 263-8555, Japan; Department of Radiation Oncology, Faculty of Medicine, University of Tsukuba, 1-1-1, Tennodai, Tsukuba, Ibaraki 305-8575, Japan; Global Center for Biomedical Science and Engineering, Faculty of Medicine, Hokkaido University, Kita 15, Nishi 7, Kita-ku, Sapporo, Hokkaido 060-8638, Japan; Department of Biostatistics, Faculty of Medicine, University of Tsukuba, 1-1-1, Tennodai, Tsukuba, Ibaraki 305-8575, Japan; Radiation and Proton Therapy Center, Shizuoka Cancer Center, 1007 Shimonagakubo, Nagaizumi, Shizuoka 411-8777, Japan; Department of Radiation Oncology, Faculty of Medicine, University of Tsukuba, 1-1-1, Tennodai, Tsukuba, Ibaraki 305-8575, Japan; Proton Therapy Center, Sapporo Teishinkai Hospital, 3-1, Kita 33, Higashi 1, Higashi-ku, Sapporo, Hokkaido 065-0033, Japan; Department of Radiation Oncology, Southern Tohoku Proton Therapy Center, 7-172, Yatsuyamada, Koriyama, Fukushima 963-8052, Japan; Department of Urology, Osaka Medical and Pharmaceutical University, 2-7, Daigakumachi, Takatsuki, Osaka 569-8686, Japan; Department of Urology, School of Medicine, Iwate Medical University, 1-1-1, Idaidori, Yahaba-Cho, Iwate 028-3694, Japan; Department of Radiation Oncology, Faculty of Medicine, Hokkaido University, Kita 15, Nishi 7, Kita-ku, Sapporo, Hokkaido 060-8638, Japan; Department of Radiation Oncology, Faculty of Medicine, University of Tsukuba, 1-1-1, Tennodai, Tsukuba, Ibaraki 305-8575, Japan

**Keywords:** muscle-invasive bladder cancer, proton beam therapy, bladder preservation therapy, chemoradiotherapy

## Abstract

To assess the safety and efficacy of proton beam therapy (PBT) for muscle-invasive bladder cancer (MIBC), we examined the outcomes of 36 patients with MIBC (cT2-4aN0M0) who were enrolled in the Proton-Net prospective registry study and received PBT with concurrent chemotherapy from May 2016 to June 2018. PBT was also compared with X-ray chemoradiotherapy in a systematic review (X-ray (photon) radiotherapy). The radiotherapy consisted of 40–41.4 Gy (relative biological effectiveness (RBE) delivered in 20–23 fractions to the pelvic cavity or the entire bladder using X-rays or proton beams, followed by a boost of 19.8–36.3 Gy (RBE) delivered in 10–14 fractions to all tumor sites in the bladder. Concurrently, radiotherapy was given with intra-arterial or systemic chemotherapy of cisplatin alone or in combination with methotrexate or gemcitabine. Overall survival (OS), progression-free survival (PFS) and local control (LC) rates were 90.8, 71.4 and 84.6%, respectively, after 3 years. Only one case (2.8%) experienced a treatment-related late adverse event of Grade 3 urinary tract obstruction, and no severe gastrointestinal adverse events occurred. According to the findings of the systematic review, the 3-year outcomes of XRT were 57–84.8% in OS, 39–78% in PFS and 51–68% in LC. The weighted mean frequency of adverse events of Grade 3 or higher in the gastrointestinal and genitourinary systems was 6.2 and 2.2%, respectively. More data from long-term follow-up will provide us with the appropriate use of PBT and validate its efficacy for MIBC.

## INTRODUCTION

Bladder cancer is a malignant tumor that develops from the bladder’s urothelial mucosa, and the age-adjusted incidence rate per 100 000 Japanese population in 2018 was 7.2 (12.6 in males and 2.8 in females). It is more common in the elderly, with >90% of patients being over the age of 60 [1]. According to the degree of invasion of the lesion, it is roughly classified as non-muscle-invasive bladder cancer (NMIBC) or muscle-invasive bladder cancer (MIBC), and the standard treatment varies greatly between the two. The treatment policy for NMIBC is to preserve the bladder through trans-urethral resection of the bladder tumor (TURBT) and intravesical instillation therapy, whereas surgical total cystectomy is the standard treatment for MIBC [2, 3]. In the past, definitive radiotherapy was rarely chosen for MIBC patients; however, in many studies, combined modality therapy (CMT) that consists of maximal TURBT followed by chemoradiotherapy in appropriately selected MIBC patients demonstrated favorable outcomes. According to recent systematic reviews, meta-analyses and propensity score matching analyses [4–6], CMT treatment outcomes are comparable to surgery. CMT is currently listed as a recommended Category 1 option in the guidelines for patients who are ineligible for radical surgery due to co-morbidities or advanced age or who wish to preserve the bladder. The majority of CMT radiotherapy is X-ray (photon) radiotherapy (XRT).

Particle therapy is a treatment method that uses accelerated particles to irradiate lesions (mainly protons and carbon ions). Because particle beams emit energy at a specific depth based on incident energy and then rapidly decay (Bragg peak phenomenon), the exit dose is reduced compared with XRT, resulting in a higher dose concentration. Takaoka *et al*. studied selective bladder-preserving therapy with proton beam therapy (PBT) for cT2-3N0M0 muscle-invasive cancer and found a 5-year cumulative overall survival (OS) rate and progression-free survival (PFS) rate of 82 and 77%, respectively. Furthermore, a favorable result of 2% late non-hematologic toxicity of Grade 3 or higher was reported [7]. It is hypothesized that the particle therapy could deliver a higher dose to the tumor without increasing the dose to nearby organs at risk, such as the small intestine and bladder. However, because there has been no randomized controlled trial comparing XRT and particle beam therapy, there is insufficient evidence to support the safety and efficacy of particle beam therapy for bladder cancer.

Particle beam therapy is covered by Japanese national health insurance for some conditions, such as pediatric tumors, bone and soft tissue tumors and localized prostate cancer, but bladder cancer is not yet covered by national health insurance and is performed as advanced medical care. Because the cost burden on patients increases significantly in advanced medical care when compared with medical care covered by health insurance, conducting a randomized controlled trial to compare XRT and particle therapy for bladder cancer is difficult. As a result, the Japanese Society for Radiation Oncology (JASTRO) began a prospective registry study in May 2016 to prospectively register all patients receiving PBT in Japan. We present the treatment outcomes of bladder cancer patients enrolled in this study through June 2018 as well as a systematic literature review to assess the utility of particle therapy for bladder cancer.

## MATERIALS AND METHODS

### Systematic examination

#### Protocol for searching

A systematic search of the PubMed online database was conducted in accordance with the Preferred Reporting Items for Systematic Reviews and Meta-Analyses guidelines [8]. Because the current bladder cancer guidelines did not mention particle therapy and no search formula was available, we used the National Institutes for Quantum Science and Technology library to search English literature on CMT for MIBC published between January 2000 and September 2020. Because PBT is the only particle beam therapy used to treat bladder cancer in Japan, only reports on PBT were searched. Table 1 displays the search terms and formulas. The systematic review team also conducted a manual search.

**Table 1:** The terms and formulae for systematic search

Modality	ID	Formula	Hit
XRT	#1	‘bladder cancer’ [TIAB]	34,858
	#2	‘cancer’ [TIAB] ‘cancers’ [TIAB] OR ‘neoplasm’ [TIAB] ‘neoplasms’ [TIAB] OR ‘neoplasms’ [MeSH Terms] OR ‘tumor’ [TIAB] OR ‘tumors’ [TIAB] OR ‘tumour’ [TIAB] OR ‘carcinoma’ [TIAB] OR ‘carcinomas’ [TIAB]	2,236,916
	#3	‘x-irradiation’ [TIAB] OR ‘x-ray irradiation’ [TIAB] OR ‘x-radiation’ [TIAB] OR ‘x-ray radiation’ [TIAB] OR ‘x-ray therapy’ [TIAB] OR ‘x-ray treatment’ [TIAB] OR ‘x-rays’ [MeSH Terms] OR ‘x-ray therapy’ [MeSH Terms] OR ‘BT’ [TIAB] OR ‘IMRT’ [TIAB] OR ‘3D-CRT’ [TIAB] OR ‘SRT’ [TIAB] OR ‘SBRT’ [TIAB] OR ‘radiation therapy’ [TIAB] OR ‘radiotherapy’ [TIAB] or ‘radiotherapy’ [MeSH Terms]	321,954
		#1 AND #2 AND #3	1375
PBT	#1	‘bladder’ [TIAB]	161,511
	#2	(((‘cancer’ [TIAB] AND ‘cancers’ [TIAB]) OR ‘neoplasm’ [TIAB]) AND ‘neoplasms’ [TIAB]) OR ‘neoplasms’ [MeSH Terms] OR ‘tumor’ [TIAB] OR ‘tumors’ [TIAB] OR ‘tumour’ [TIAB] OR ‘carcinoma’ [TIAB] OR ‘carcinomas’ [TIAB]	2,227,323
	#3	‘proton therapy’ [TIAB] OR ‘proton radiotherapy’ [TIAB] OR ‘proton beam therapy’ [TIAB] OR ‘proton beam radiotherapy’ [TIAB] OR ‘carbon ion therapy’ [TIAB] OR ‘carbon ion radiotherapy’ [TIAB] OR ‘carbon ion beam therapy’ [TIAB] OR ‘carbon ion beam radiotherapy’ [TIAB] OR ‘heavy ion radiotherapy’ [TIAB] OR ‘heavy ion radiotherapy’ [MeSH Terms] OR ‘hadron therapy’ [TIAB] OR ‘hadrontherapy’ [TIAB]	6890
		#1 AND #2 AND #3	135

TIAB = title/abstract, MeSH = medical subject headings.

#### Article selection criteria

The population, intervention, comparison and outcome (PICO) framework was used to define the inclusion criteria for the literature. We defined the population as MIBC without metastasis (Clinical Stages II and III, T2-4aN0M0 according to the seventh edition of the UICC TNM classification); the intervention as PBT with concurrent chemotherapy; the comparison as XRT with concurrent chemotherapy and the outcome as OS rate, PFS rate, local control (LC) rate, bladder preservation rate (BPR) and frequency of urogenital (GU) and gastrointestinal (GI) Grade 3 or higher adverse events. Because we expected to find a limited number of articles on XRT and PBT for MIBC, we decided to include both retrospective and prospective clinical trials that reported at least one of the PICO outcomes. When multiple publications from a single institution were reported, only the most recent or most relevant study’s outcome data were included. The number of patients required for each study was set at 10. Case reports, proceedings, preclinical studies and review articles were not considered.

#### Screening

As part of the primary screening, two radiation oncologists from the systematic review team independently checked the titles and abstracts of the extracted literature and chose articles that met the selection criteria. The full texts of the articles chosen after the primary screening were checked as part of the secondary screening, and only those that met the selection criteria were accepted. Documents where there was a disagreement about inclusion or exclusion were resolved through team discussions.

#### Data extraction

A group of two members of the systematic review team extracted data. A third member was consulted to resolve the discrepancy (H.I. or M.A.). When OS, PFS and LC were not explicitly stated in the text, they were calculated based on the total number of patients, the size of the risk set at each time point and the censoring rate. The following parameters were extracted from the articles: number of patients, age, gender, clinical stage, OS rates at 1–3 years, PFS rates at 1–3 years, LC rates at 1–3 years, CMT, BPR, complete response rate and the frequency of Grade 3 or higher GU and GI adverse events.

### Registry data analysis

#### Patients

From May 2016 to June 2018, patients with primary bladder cancer were prospectively enrolled in the ‘Proton-Net’ Japanese proton therapy multicenter database system in which a total of 13 facilities are participating. Patients with histopathologically or clinically diagnosed MIBC without metastasis at any site are eligible for inclusion in PBT as advanced medical care in Japan. Therefore, the inclusion criteria for this study were patients diagnosed with urothelial cell carcinoma, MIBC (cT2-4aN0M0), who had undergone PBT with concurrent chemotherapy as a curative treatment, and who had no other concurrent cancer irrespective of age and performance status. Computed tomography and bone scintigram were commonly used for staging, but positron-emission tomography was not mandatory.

#### Treatment

PBT was carried out using a treatment protocol based on the unified treatment policy established by the urologic cancer working group subcommittee of the Japanese Society of Radiation Oncology’s Particle Therapy Committee (JASTRO). The relative biological effectiveness (RBE) value of 1.1 was used for the PBT treatment planning. Following 40–41.4 Gy (RBE) irradiation of the pelvic cavity or the entire bladder in 20–23 fractions with proton beams or X-rays, local lesions were boosted with proton beams using the following treatment protocol: (i) the treatment protocol for cases not adjacent to the GI tract was 33–36.3 Gy (RBE) in 10–11 fractions to all tumor sites in the bladder (total dose of 73.0–77.7 Gy (RBE) in 30–34 fractions) and (ii) for cases near the GI tract, the treatment protocol was 19.8–25.2 Gy (RBE) in 11–14 fractions (total dose of 59.8–66.6 Gy (RBE) in 31–37 fractions). As a dose constraint for organ at risk, the total dose to 2 cc of the intestine was allowed up to 60 Gy (RBE) in equivalent dose in 2 Gy fraction. At every treatment session of proton boost, the bladder was filled with urine or sterilized water to keep moderately full bladder.

Since there is no clear evidence of prophylactic pelvic irradiation for MIBC, the decision to perform pelvic irradiation was left to the discretion of the facility. In case pelvic irradiation was administered, the four-field technique of anteroposterior–posteroanterior and opposing lateral fields was used. Each field was set to include the tumor, the bladder and pelvic lymph nodes (internal iliac, external iliac, obturator and presacral lymph nodes). Proton beams were not used for pelvis irradiation in all patients because of the limitations on the field size. For X-ray therapy, high-energy X-rays of ≥6 MV energy were commonly used.

#### Endpoints and statistical analysis

OS, PFS, LC and treatment-related Grade 3 or higher late toxicity after proton therapy for MIBC were all evaluated in this study.

CT scans and urine cytology were examined every 3–4 months for 2 years after treatment and every 3–6 months thereafter, in accordance with the bladder cancer study's follow-up policy, with additional cystoscopy and biopsy performed if bladder recurrence was suspected.

The time span between the first day of PBT and the date of death or the last follow-up visit was defined as the follow-up period. OS defined an event as death from any cause. PFS defined an event as death from any cause or any signs of cancer progression. In the case of LC, an event was defined as any sign of superficial or invasive recurrences in the bladder. The time from the start of PBT to the date of the events or the last follow-up visit was used to calculate the survival function for each outcome using the Kaplan–Meier method and log-rank test. A *P*-value of <0.05 was considered to be statistically significant. The treatment-related late toxicity of Grade 3, defined as complications occurring 3 months after the completion of PBT, was assessed using the Common Terminology Criteria for Adverse Events (version 4.0). Prior approval was obtained from each center's ethics committee for this multicenter database prospective registry study, and all patients provided written informed consent in accordance with the Helsinki Declaration. This study has been registered with the UMIN-clinical trials registry under the study number UMIN000022917.

## RESULTS

### Systematic review

**Fig. 1:**
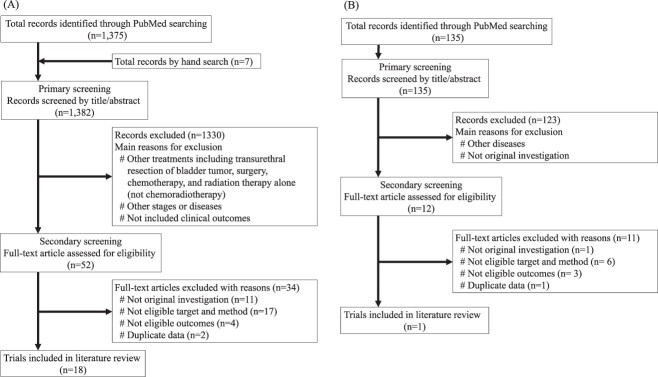
Flow diagrams of the selection process for X-ray radiotherapy (**A**) and PBT (**B**).

A flow diagram that outlines the selection process is shown in Fig. 1A (XRT) and Fig. 1B (PBT).

A total of 1375 articles on XRT were extracted via systematic search, with an additional 7 articles added via hand search. Primary screening excluded 1330 articles, and secondary screening was performed on the remaining 52. Thirty four of these were eliminated for the reasons outlined in Fig. 1A, and 18 were eventually chosen [9–26].

In terms of PBT, a systematic search yielded 135 articles. In the primary screening, 123 articles were eliminated, and the remaining 12 articles were subjected to secondary screening. Only two articles reported clinical outcomes, and because they were from the same institution and overlapped in the analysis subjects, only one was chosen [7]. We determined that meta-analysis was impossible due to the scarcity of articles reporting the results of PBT.

Table 2 provides a summary of the articles chosen. The 3-year OS, PFS and LC outcomes of XRT were 57–84.8%, 39–78% and 51–68%, respectively. The frequency of Grade 3 or higher adverse events ranged from 0 to 41% for GU and from 0 to 9.1% for GI, with weighted means of 6.2 and 2.2%, respectively. On the other hand, PBT demonstrated 90% in OS and 80% PFS after 3 years, with the frequency of GU and GI Grade 3 or higher adverse events being 2.9 and 0%, respectively.

**Table 2:** Summary of literature review of XRT and PBT for bladder cancer

Author (year)	Number of patients	Study type	RT dose (Gy/fraction)	Chemotherapy method	3-year OS (%)	3-year PFS (%)	3-year LC (%)	BPR (%)	CR (%)	≥G3 toxicity (GU/GI)
XRT										
Peyromaure (2004) [9]	43	Retrospective	48 Gy/16 fr	Systemic		63		74.4 (OA)	74.4	0%/7.0%
Kragelj (2005) [10]	84	Phase II	63.8–64 Gy/32–35 fr	Systemic	57		61	NA	78	41%/6.4%
Gogna (2006) [11]	113	Phase II	63–64 Gy/32–35 fr	Systemic		39	51	61 (OA)	70	4%/2%
Hashine (2009) [12]	94	Retrospective	36–60 Gy/18–30 fr	Intra-arterial	81			89.7/87.6 (5y/10y)	89	Total 6.4%
Efstathiou (2009) [13]	157	Prospective cohort	Varied	Systemic				NA	NA	5.7%/1.9%
Lin (2009) [14]	30	Phase II	64.8 Gy/36 fr	Systemic	77	54		NA	77	4.5%/0%
Sabaa (2010) [15]	104	Phase II	60–65 Gy/30–36	Systemic					78.8	0%
Lagrange (2011) [16]	53	Phase II	63 Gy/35 fr	Systemic	63		57	67 (8y)	NA	NA
Choudhury (2011) [17]	50	Phase II	52.5 Gy/20 fr	Systemic	75			NA	88	2%/2%
Tunio (2012) [18]	102: WP 98: local	Randomized study	65 Gy/35fr	Systemic	81 75	78 73		58.9 (OA) 57.1 (OA)	93.1 92.8	2%/1% 1%/0%
James (2012) [19]	182	Randomized study	55 Gy/20 fr or 64 Gy/32 fr	Systemic	56	63		88 (2y)	NA	8.3% (OA)
Efstathiou (2012) [20]	348	Prospective cohort	Varied	Systemic	64			80 (5y)	72	NA
Zapatero (2012) [21]	80	Prospective cohort	Varied	Systemic	75 73				74	6.3%/2.5%
Mitin (2013) [22]	46: PTX 47: 5FU	Randomized study	64.3 Gy/42fr	Systemic	79 81				72 62	6.5%/2.2% 4.3%/0%
Lee (2014) [23]	70	Retrospective	59.4 Gy/33 fr (Median)	Systemic	57			NA	78.1	1.4%/0%
Yoshioka (2016) [24]	134	Retrospective	60 Gy/30 fr	Intra-arterial	80		68	90 (3y)	80	6%/2%
Coen (2019) [25]	33: FP 33 GEM	Randomized study	64.3 Gy/42fr 64 Gy/32fr	Systemic	81.8 84.8			90.9 (3y) 84.8 (3y)	88 78	6.0%/6.1% 6.1%/9.1%
Gofrit (2020) [26]	105	Retrospective	60–66 Gy/30–33 fr	Systemic				92.4 (OA)	NA	NA
PBT										
Takaoka (2017) [7]	70	Retrospective	77.7 Gy(RBE)/34 fr (X-ray 41.4 Gy/23 fr + proton 36.3 Gy(RBE)/11 fr)	Intra-arterial	90	80		93 (OA)	NA	2.9%/0%

Abbreviations: WP = whole pelvis, OA = overall, BPR = bladder preservation rate, CR = complete response rate, GU = genitourinary, NA = not assessed, PTX = paclitaxel, 5FU = 5-fluorouracil, FP = cisplatin plus 5FU, GEM = gemcitabine.

### Registry data analysis

Of 43 MIBC patients who were registered in Proton-Net, 36 patients from three facilities were studied. Tables 3 and 4 summarize the patient characteristics. The median age of these patients was 70.5 years (range: 48–87), and the median follow-up period was 41.4 months (range: 3.2–54.9).

**Table 3:** Characteristics of the patients with MIBC from the registry dataset

	Median	Range
Age (years)	70.5	48–87
<71	18	(50.0%)
≥71	18	(50.0%)
Sex		
Male	28	(77.8%)
Female	8	(22.2%)
ECOG performance status		
0	27	(75.0%)
1	7	(19.4%)
2	2	(5.6%)
Histopathological diagnosis		
Urothelial cell carcinoma	36	(100%)
Unknown	0	(0%)
T stage		
T2a	4	(11.1%)
T2b	20	(55.6%)
T3a	1	(2.8%)
T3b	10	(27.8%)
T4a	1	(2.8%)
	Median	Range
Tumor size (cm)	3.0	1.0–5.9
<5.0	33	(91.7%)
≥5.0	3	(8.3%)
Hydronephrosis		
+	4	(11.1%)
−	32	(88.9%)
Lesions		
Solitary tumor	26	(72.2%)
Multifocal tumor	10	(27.8%)
Operability		
Operable	30	(83.3%)
Inoperable	6	(16.7%)
The status of bladder cancer	3	
Other medical reasons	3	

ECOG = Eastern Cooperative Oncology Group.

**Table 4:** Characteristics of treatment from the registry dataset

Protocol	
73–77.7 Gy(RBE) in 30–34 fractions	26 (72.2%)
59.8–66.6 Gy(RBE) in 30–37 fractions	9 (25.0%)
Other:70 Gy(RBE) in 35 fractions	1 (2.8%)
Pelvic irradiation	
Yes	33 (91.7%)
No (whole-bladder irradiation)	3 (8.3%)
Chemotherapy	
Intra-arterial chemotherapy	20 (55.6%)
CDDP (50 mg/m^2^) + MTX (30 mg/m^2^), every 3 weeks (2–3 courses)	18
CDDP (50 mg/m^2^), every 3 weeks (2–3 courses)	2
Systemic chemotherapy	16 (44.4%)
CDDP (100 mg/m^2^) + GEM (1200 mg/m^2^), every 3 weeks (2–3 courses)	1
CDDP (30 mg/m^2^), weekly (5 courses)	15

CDDP = cisplatin, MTX = methotrexate, GEM = gemcitabine.

According to the protocol, 27 patients received a total dose of 73–77.7 Gy (RBE) in 30–34 fractions, but 9 patients received a total dose of 59.8–66.6 Gy (RBE) in 30–37 fractions because their lesions were located close to the GI tract. The other patient was initially treated using the protocol close to the GI tracts. However, additional irradiation was required due to a suspected residual tumor, resulting in a final dose of 70 Gy (RBE) in 35 fractions. In 33 of the 36 patients, pelvic irradiation with X-ray was performed; 3 patients received whole-bladder irradiation with PBT alone, which was followed by PBT boost to the lesion sites in all cases. Twenty patients were given intra-arterial chemotherapy, while the remaining 16 were given systemic chemotherapy.

**Fig. 2:**
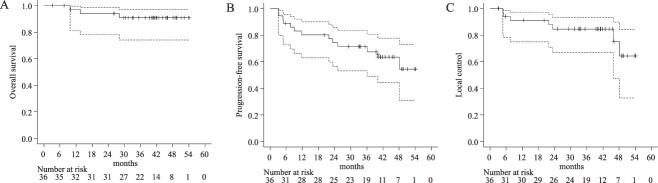
Kaplan–Meier curves showing the probabilities of OS (**A**), PFS (**B**) and LC (**C**). The solid line indicates the estimated value, and the upper and lower dotted lines represent the upper and lower limits of 95% confidence interval, respectively.

Recurrence was observed in 13 patients (36.1%), with 7 patients (19.4%) having local recurrences and 6 patients (16.7%) having metastatic lesions. The data entered from the registry data showed four patients with distant metastasis and two with regional lymph node metastasis. At the time of the last observation, three patients (8.3%) were dead; two died of cancer progression, while the other died of intercurrent disease with no sign of recurrence. After local recurrence in the bladder, one patient underwent radical cystectomy. The 3-year OS, PFS and LC rates of the 36 patients in this study were 90.8% (95% CI: 74.0–96.9), 71.4% (95% CI: 53.3–83.5) and 84.6% (95% CI: 66.8–93.3), respectively (Fig. 2). In univariate analysis, operability, the presence of hydronephrosis and prophylactic pelvic irradiation were factors associated with OS (*P* = 0.010, <0.001, <0.001, respectively), while the presence of hydronephrosis, T stage and prophylactic pelvic irradiation were factors associated with PFS (*P* = 0.044, =0.006, <0.001), but there were no factors associated with LC (Table 5).

**Table 5:** Univariate analyses of risk factors for progression

			OS			PFS			LC		
		*n*	1 year	3 years	*P*-value	1 year	3 years	*P*-value	1 year	3 years	*P*-value
Operability											
Operable		30	100.0%	96.2%	**0.010**	86.5%	76.2%	0.263	96.4%	89.0%	0.275
Inoperable		6	83.3%	66.7%		50.0%	50.0%		66.7%	66.7%	
Hydronephrosis											
Negative		32	100.0%	96.4%	**<0.001**	84.2%	77.4%	**0.044**	93.2%	86.0%	0.390
Positive		4	75.0%	50.0%		50.0%	25.0%		75.0%	75.0%	
Lesion											
Solitary		26	96.0%	92.0%	0.784	80.8%	68.7%	0.570	92.0%	83.2%	0.646
Multiple		10	100.0%	87.5%		80.0%	80.0%		88.9%	88.9%	
Tumor size											
<5.0 cm		33	96.8%	89.8%	0.574	84.7%	75.0%	0.168	93.5%	86.4%	0.351
≥5.0 cm		3	100.0%	100.0%		33.3%	33.3%		66.7%	66.7%	
T stage											
T2a–T3a		25	100.0%	95.7%	0.084	91.8%	83.1%	**0.006**	91.8%	83.1%	0.548
T3b–T4a		11	88.9%	77.8%		54.5%	45.5%		88.9%	88.9%	
Prophylactic pelvic irradiation											
+		33	100.0%	96.4%	**<0.001**	87.8%	78.0%	**<0.001**	93.4%	86.5%	0.168
−		3	66.7%						66.7%		
Intestines											
Not close		26	100.0%	95.5%	0.124	84.4%	76.4%	0.542	95.8%	91.5%	0.120
Close		10	90.0%	80.0%		70.0%	58.3%		80.0%	66.7%	

Significant P-values are shown in bold typeface.

Only one case (2.8%) experienced Grade 3 urinary tract obstruction as a result of treatment, but no severe GI adverse effects were observed. The Grade 1–2 toxicities data were not collected in Proton-Net and, therefore, are unavailable.

## DISCUSSION

Using prospective nationwide registry data, this study assessed the clinical efficacy of PBT as a CMT that consists of maximal TURBT, which was followed by chemoradiotherapy as a definitive bladder preservation therapy for patients with Stages II and III MIBC. The 3-year OS and PFS rates in the study were 90.8 and 71.4%, respectively, and no Grade 3 or severe GI adverse effects were observed. The results of the largest study on PBT for MIBC were previously published by a Tsukuba group, and the outcomes of 70 Stages II and III MIBC patients treated with CMT using PBT until 2015 (before the start of the nationwide registry) were reported [7]. In their study, the 3-year OS and PFS rates were 92 and 80%, respectively, with no Grade 3 GI toxicity. As a result, the prospective registry data in this study appear to replicate the clinical outcomes of feasibility and efficacy reported in the previous study.

There have been no randomized trials comparing PBT to XRT as CMT for patients with Stages II–III MIBC. As a result, we attempted SR to compare the clinical outcomes of the treatments. As a result of our systematic review, the 3-year outcomes of XRT-based CMT were 57–84.8% in OS, 39–78% in PFS and 51–68% in LC, and Grade 3 or higher GI adverse events ranged from 0 to 9.1%, with a weighted mean of 2.2% (Table 2). The dose of irradiation to bladder tumors is critical in radiotherapy to completely eradicate tumor cells, but it is known that there are dose dependencies in the severity and incidence of late adverse effects on adjacent organs at risk. The proton beams have a physical property in radiotherapy for deep-sheeted tumors. While X-rays exhibit the characteristic of depth dose buildup, proton beams can be stopped at a specific depth in the body to impart a maximal radiation dose to the target during treatment. Furthermore, because they can be stopped at a specific depth in the body during treatment, a favorable dose distribution of the proton beams can be obtained by smaller numbers of beam ports than used for X-rays and that could explain why we obtained favorable results regarding the toxicity outcomes of prospective registry data in previous and current studies of PBT for MIBC [27, 28]. As a result, PBT can deliver sufficient dose to control the bladder tumor without increasing the risk of adverse events in normal tissues such the GI tracts and the normal bladder. PBT’s safety in treating patients with Stages II–III MIBC may thus be confirmed.

In terms of the efficacy of using protons in CMT for MIBC, this study found that the 3-year OS and LC rates after PBT were ~90%, which appears to be superior to other outcomes after CMT using X-rays. In this study, seven (19.4%) patients experienced local recurrence, and the recurrence rate appears to be lower than in previous XRT-based CMT protocols (ranging from 11 to 43%) [29]. Furthermore, the previous and our studies’ 3-year LC rates of PBT were 80 and 84.6%, respectively, but the corresponding rate after XRT-based CMT ranged from 51 to 68% (Table 2). Because dose escalation is a reasonable approach to bladder tumor control, the higher irradiation dose (77.7 Gy (RBE)) given by PBT compared with the standard dose (60–70 Gy) in other studies may contribute to the high LC rate [6–26]. In this study, patients were treated with two protocols, a high-dose group and a low-dose group, depending on whether the lesion was close to the GI tract. However, this analysis did not confirm an oncologic outcome advantage in the high-dose group, which is farther from the GI tract. Since prior studies have suggested improved LC with increasing doses, the reason this difference was not significant in the present analysis may be due to the small number of cases. This study included T3b–T4 diseases (30.6%), multifocal tumors (27.8%) and the presence of tumor-related hydronephrosis (11.1%), all of which have been identified as risk factors for recurrence after CMT in Stages II–III MIBC patients [29]. Similarly, in this analysis, hydronephrosis was associated with OS and PFS, and T stage with PFS. On the other hand, tumor volume and size are known to be significant predictors of tumor control [29, 30], and the median diameter of the bladder tumors in the study was 3.0 cm (ranging from 1.0 to 5.9 cm). As a result, although the effectiveness of proton beams on CMT for MIBC may be confirmed, the relatively small size of tumors may influence our preferable results. In this analysis, some factors (especially, tumor size and pelvic irradiation) had very small sample sizes for each stratum, so the analysis results should be interpreted carefully.

The usefulness of PBT for various urological cancers has been confirmed, particularly in the step-by-step treatment of prostate cancer [31–34], but publications on PBT studies for bladder cancer have only come from a Tsukuba group [7, 27, 35]. As a result, SR for PBT for MIBC in this study must be immature. Despite this significant limitation, the California Protons Cancer Therapy Center and the Texas Center for Proton Therapy, as well as a number of Japanese PBT institutes, have begun to use protons for MIBC [36]. Although randomized trials or matched-pair analyses are required to validate the efficacy of PBT, a rapid increase in the number of PBT facilities around the world will be able to alleviate the concern in the future. Furthermore, there are some limitations to our research. Because the nationwide registry was begun in 2016, and only patients treated in the first 2 years were analyzed in the study, the number of patients in the obtained data was relatively small (*n* = 37). However, PBT was performed on all MIBC patients in accordance with JASTRO's treatment policy, and data from them were collected prospectively. As a result, we will be able to obtain more reliable and larger registry data in the coming years. Second, patient and treatment characteristics, such as chemotherapy regimen and use of prophylactic nodal irradiation, were inconsistent, despite the fact that a previous Japanese multi-institutional study found that the type of chemotherapy administered had no effect on LC or OS rates [37]. Future research using the continuous nationwide registry will address these issues and provide a detailed indication and treatment method for PBT for MIBC.

## CONCLUSION

PBT is expected to provide at least the same toxicity as XRT-based CMT for Stages II-III MIBC while improving survival. More detailed data from long-term follow-up will provide us with an appropriate use of protons and will validate the efficacy of PBT as a CMT for MIBC.

## CONFLICT OF INTEREST

None.

## FUNDING

This work was supported by Hokkaido University (Functional enhancement promotion expenses by the Ministry of Education, Culture, Sports, Science and Technology) and AMED under Grant Number JP16lm0103004.

## ACKNOWLEDGEMENT

The authors would like to thank Enago (www.enago.jp) for the English language review.

## DATA AVAILABILITY

The datasets generated during and/or analyzed during the current study are available from the corresponding author on reasonable request.

## PRESENTATION AT A CONFERENCE

None.

## CLINICAL TRIAL REGISTRATION NUMBER

None.
